# Supplementary material for: Chromothripsis during telomere crisis is independent of NHEJ, and consistent with a replicative origin
Source: Genome Res. 2019 May;29(5):737–49. doi: 10.1101/gr.240705.118 (PMC6499312; doi:10.1101/gr.240705.118)
Supplement: Supplemental Material [file supp_gr.240705.118_Supplemental_file_1.zip › contigs/annotated_contigs/DB46/contig.2.DB46_length_479_mean_cov_5.63674321503.docx]

**DB46_length_479_mean_cov_5.63674321503**

```
GCCAGGCGTAGTGGCGGGCA|CCTGTAATCCCAGCTACTCGGGAGGCTGAGGCAGGAGAATTGCTCGAACCTGGGAGACGGAGGTTGCA
                     >chr5:162348483-162348818 - E=5e-160
GTGAGCCGAGATTGAGCCATTGCACTCCAGCCTGGGCAATGAGCAAAACTCTGTCTCAAAAACAAACAAACAAAAATCACAAGATTCAA

CTCTATGCTGTCTACAAGTAACTAATTTTAGATTTTAAGACACACACTCTGGTTGTTAAAGGATGGAGAAGATATCCCATGAAATGAGA

ACAACAACAGCATAAAGGAGCAGAATGGCTATACTTATATCACACAAAACAGATTTTCAATCAAAACTGTCACAATACACAAAGGGAA|

A|ACTAAAAAGTGCAGCAAAAACTAAGGCCAAAATTAGCACATGAGAGAAAATAATAAAGACTAGAGCAGAAACAGACAAATGAGACTA
>chr5:162347675-162347800 - E=1e-60
GAGAAACAATAGAAAAATCAATGACACTAAGAGTTGG
```
